# Formation and Biopharmaceutical Characterization of Electrospun PVP Mats with Propolis and Silver Nanoparticles for Fast Releasing Wound Dressing

**DOI:** 10.1155/2016/4648287

**Published:** 2016-02-14

**Authors:** Erika Adomavičiūtė, Sigitas Stanys, Modestas Žilius, Vaida Juškaitė, Alvydas Pavilonis, Vitalis Briedis

**Affiliations:** ^1^Department of Materials Engineering, Faculty of Mechanical Engineering and Design, Kaunas University of Technology, Studentų 56, LT-51424 Kaunas, Lithuania; ^2^Department of Clinical Pharmacy, Faculty of Pharmacy, Lithuanian University of Health Sciences, Sukilėlių pr. 13, LT-50161 Kaunas, Lithuania; ^3^Institute of Microbiology and Virology, Lithuanian University of Health Sciences, Eivenių g. 4, LT-50161 Kaunas, Lithuania

## Abstract

Antibacterial, antiviral, antifungal, antioxidant, anti-inflammatory, and anticancer activities of propolis and its ability to stimulate the immune system and promote wound healing make it a proper component for wound dressing materials. Silver nanoparticles are recognized to demonstrate strong antiseptic and antimicrobial activity; thus, it also could be considered in the development of products for wound healing. Combining propolis and silver nanoparticles can result in improved characteristics of products designed for wound healing and care. The aim of this study was to formulate electrospun fast dissolving mats for wound dressing containing propolis ethanolic extract and silver nanoparticles. Produced electrospun nano/microfiber mats were evaluated studying their structure, dissolution rate, release of propolis phenolic compounds and silver nanoparticles, and antimicrobial activity. Biopharmaceutical characterization of electrospun mats demonstrated fast release of propolis phenolic compounds and silver nanoparticles. Evaluation of antimicrobial activity on* Staphylococcus aureus*,* Staphylococcus epidermidis*,* Enterococcus faecalis*,* Escherichia coli*,* Pseudomonas aeruginosa*,* Proteus vulgaris*,* Bacillus subtilis*,* Bacillus cereus*, and* Candida albicans* strains confirmed the ability of electrospun mats to inhibit the growth of the tested microorganisms.

## 1. Introduction

Current advances in formulation of novel drug delivery systems offer a great opportunity to develop new therapies or to enhance the effectiveness of available medical treatments. These advances are particularly relevant to the field of regenerative medicine, including challenging healthcare issues in wound healing and skin repair [1]. New applications of biotextiles have been identified after the development of nanofiber manufacturing technologies. Combination of nanotechnology and biotextile technology has resulted in formation of bionanotextiles. Currently, bionanotextiles are used in the development of wound dressings, bandages, and tissue scaffolds [2]. Electrospinning is considered as a dominating technique in manufacturing of biotextiles. A wide range of polymers can be formulated into continuous and functional nano/microfibers by a straightforward electrospinning technique. The surface of a polymer solution droplet is electrically charged by applying high-voltage electrostatic field, thereby inducing the ejection of a liquid jet through a spinneret. On the way to the collector, the jet is subjected to forces that allow it to stretch. Simultaneously, the jet solidifies due to solvent evaporation, and electrically charged nano/microfibers are formed [3]. Polyvinyl alcohol, chitosan, polyurethane, poly(lactide-co-glycolic acid), silk fibroin, polyvinylpyrrolidone (PVP), cellulose acetate, and some other polymers have been used in production of nano/microfibers by electrospinning to formulate wound dressings and similar products [2, 4–14]. The produced systems contained biologically active substances, products of natural origin, or antibacterial properties exhibiting nanoparticles.

Fast disintegration and release of nonsteroidal anti-inflammatory drug meloxicam have been achieved by incorporating it into PVP-based electrospun mat produced by electrospinning [4]. PVP electrospun mats have been formulated with another nonsteroidal anti-inflammatory drug indomethacin, which is indicated for pain relief and inflammation in rheumatic diseases, sprains, strains, and backache. The encapsulation efficiency was 75% w/w, with complete drug release in 45 min [5]. Electrospinning has been used for production of wound dressing using thermoresponsive poly(N-isopropylacrylamide), egg albumen, and poly(*ε*-caprolactone) blend solutions with drug gatifloxacin hydrochloride, and high antibacterial activity has been demonstrated at very low concentrations of an active substance [15]. The effect was referred to initial rapid release of gatifloxacin hydrochloride from nanofibers, which lasted up to 10 h, followed by slow and controlled release for 696 h supporting* in vitro* cell viability and* in vivo* wound healing. Similarly, electrospun nanofibrous mats for wound dressing application have been successfully produced from polycaprolactone, cellulose acetate, and dextran blend solution, incorporating a small amount of antibacterial drug tetracycline hydrochloride, that resulted in improved cell proliferation and enhanced blood clotting ability and cell attachment as well as antimicrobial activity of the composite mat [16]. Composite nanofibrous mats for wound dressing have been fabricated using blends of chitosan, gelatin, and shape memory polyurethane using an electrospinning technique and subsequent posttreatment with a silver nitrate solution. The produced mats demonstrated a shape memory effect, desirable water vapor transmission ratio, surface wettability, and antibacterial activity against the common Gram-negative and Gram-positive bacteria, cytocompatibility with fibroblasts, and necessary hemostatic characteristics. Such electrospun composite nanofibrous mats can be used as potential smart wound dressings [7].

Antibacterial protection is a necessary prerequisite for efficient wound healing. Antibacterial agents should be incorporated into wound dressing materials to prevent bacterial colonization and subsequent wound infections [6, 17]. Silver is one of the most powerful antiseptic materials available naturally, demonstrating low toxicity toward mammalian tissues. Silver nanoparticles (Ag NPs) with a diameter ranging from 10 to 100 nm exhibit a strong antibacterial effect against both Gram-positive and Gram-negative bacteria. Zein electrospun nanofibers loaded with Ag NPs of 20 nm diameter showed good antibacterial activity.* In vitro* cell tests indicated that electrospun zein mats had good bioactivity and supported cell growth and proliferation as well as adhesion [17, 18]. Electrospinning of nanofibrous polyurethane mats has been used to incorporate Ag NPs and badger oil, which is used as a traditional medicine to heal wounds [19]. Polyacrylonitrile electrospun nanofibers loaded with Ag NPs have exhibited strong antimicrobial activities against Gram-negative* Escherichia coli*, Gram-positive* Staphylococcus aureus*, and fungus* Monilia albicans* [8]. Chitosan/poly(vinyl alcohol)/Ag NP electrospun nanofibers have demonstrated capability of continuous Ag release to exhibit antibacterial activity during 16 days. These biocomposite nanofibers have shown a pronounced antibacterial activity against* Staphylococcus aureus* and* Escherichia coli* [9]. Inclusion of Ag NPs into nanofibers forming mixture can result in high antibacterial activity of the product, improved electrospinnability, and decreased diameter of electrospun nanofibers [20–22]. Complex natural products such as plant extracts, honey, and propolis with inherent biological activity have also been incorporated into electrospun nanofiber mats [23–25]. Nanofibers produced from polyvinyl alcohol cospun with honey and chitosan exhibited enhanced antibacterial activity against* S. aureus* but poor antibacterial activity against* E. coli*. The developed nanofibers with high concentrations of honey and chitosan hold the potential as effective biocompatible wound dressings [23]. Mouth-dissolving PVP fibers loaded with propolis have been produced by the electrospinning technique, and their antibacterial activity has been confirmed [24]. Biocompatible propolis loaded polyurethane nanofibers have been prepared using electrospinning of propolis/polyurethane blend solution. Incorporation of small amounts of propolis into polyurethane matrix improved the hydrophilicity and mechanical strength of electrospun mats and improved their biocompatibility. Moreover, composite nanofibers have been proved to be effective against* E. coli* [25].

The aim of this study was to produce and evaluate biopharmaceutical characteristics and antimicrobial activity of electrospun fast releasing antibacterial mats for wound dressings. The structure of electrospun mats, release kinetics of propolis components and Ag NPs, and antimicrobial activity of electrospun PVP mats were evaluated to determine their potential for application in wound healing. Water soluble polymer was chosen to achieve fast disintegration, dissolution, and release of biologically active substances, promoting epithelization and ensuring antibacterial protection. These arguments supported choice and testing of PVP, which is hygroscopic in nature, induces water absorption up to 40% of its weight under atmospheric conditions, and demonstrates appropriate biocompatibility characteristics adequate for biomedical applications [4, 26]. Combination of propolis and Ag NPs was chosen due to available information on biological activity of the abovementioned materials. Integration of propolis and Ag was expected to result in enhanced wound healing activity due to propolis anti-inflammatory, antimicrobial effects along with a broad-spectrum antimicrobial activity of Ag NPs [27].

## 2. Materials and Methods

### 2.1. Materials for Electrospinning Solution

PVP (Sigma Aldrich; Mw, 1300 000 g/mol) was used in electrospinning of mats.

Propolis ethanolic extract (PEE) was produced by using 70% ethanol aqueous solution for extraction of raw propolis under mixing with a magnetic stirrer IKAMAG® C-MAG HS7 (IKA-Werke GmbH & Co.KG, Staufen, Germany) for 1 h at room temperature. The stirrer rotation speed was 250 rpm. The ratio of raw propolis to extractive agent was 1 : 10. PEE was filtered through a paper filter with 20 to 25 *μ*m pores (DP 411, Albet® Filtration & Separation Technology, Spain) and standardized by dilution to an appropriate concentration of total phenolic compounds (750 or 1500 *μ*g/mL).

PVP polymer was dissolved in ethanol and PEE for 7 h under stirring. The concentration of PVP in PEE was 6% (mean viscosity of PVP solution was 100 mPa·s) or 8% (mean viscosity of PVP solution was 160 mPa·s).

Colloidal solution of Ag nano/microparticles was prepared by dissolving 10 g of PVP (Sigma Aldrich; Mw, 40 000 g/mol) in 80 mL of ethanol, and after full dissolution, 2 g of AgNO_3_, 10 mL of water, and 100 mL of ethanol were added to the prepared PVP solution.

### 2.2. Electrospinning Process of PVP with Propolis and Silver Particles Mats

**Figure 1:**
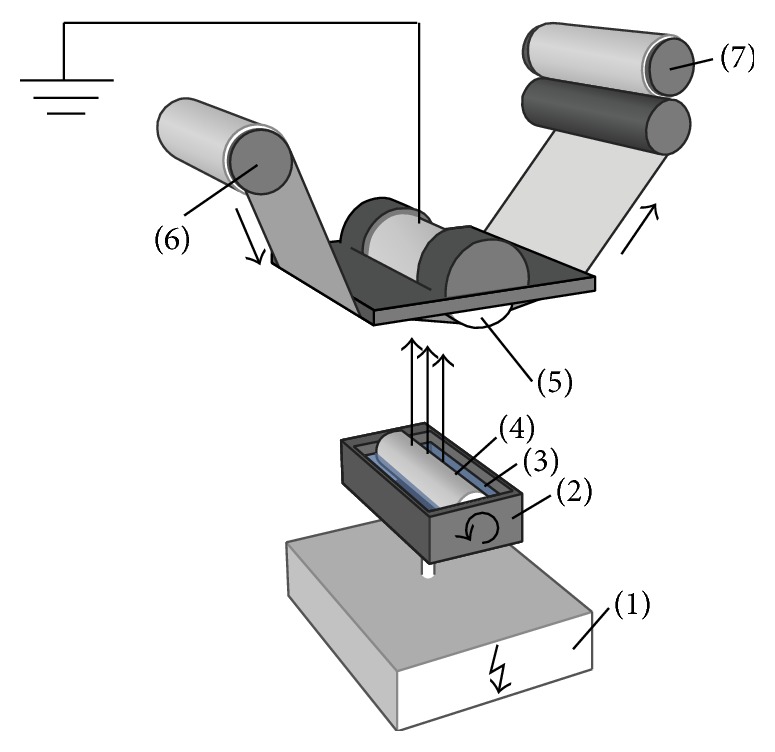
Scheme of electrospinning apparatus “Nanospider” [28]. 1: applied voltage supply, 2: bath, 3: polymer solution, 4: rotating electrode, 5: grounded electrode, 6: support material (spunbond from polypropylene fibers), and 7: support material, covered with layer of electrospun mat.

Electrospun mats were formed with roller rotating electrospinning equipment “Nanospider*™*” (Elmarco, Czech Republic). The general scheme of the apparatus is presented in Figure 1. Roller electrode 4 is rotating in bath 2 containing polymer solution 3. Polymer jets from Taylor cones are formed when created electrostatic forces exceed the surface tension of polymer solution. Polymer jets split to nano/microfibers while moving to grounded electrode 5 covered by support material 6. The nano/microfiber mat is formed and accumulated on support material 7.

Electrospun mats from PVP nano/microfibers were formed by applying 30 kV voltage; the distance between electrodes (4 and 5) was 13 cm; environmental temperature, 21°C  ± 2°C; and humidity, 60%  ± 5%.

The compositions of PVP solutions used in the production of nano/microfiber mats by electrospinning are shown in Table 1.

### 2.3. Structure Analysis of Electrospun PVP Mats with Propolis and Silver Particles

The structure of electrospun mats was confirmed by a scanning electron microscope SEM S-3400N (Hitachi, Japan) by applying 10 000-fold (5 *μ*m scale) and 1000-fold (50 *μ*m scale) magnification. The diameter of nano/microfibers was evaluated by Lucia Image 5.0 software by analyzing SEM images and measuring 200 fragments of nano/microfibers under 10 000-fold (5 *μ*m scale) magnification.

A scanning electron microscope (Hitachi S-3400N) with an energy dispersive X-ray spectrometer (Bruker Quad 5040) was used to investigate the composition of electrospun mats.

An infrared absorption spectrum was obtained by using a Fourier transform infrared spectrometer FT-IR Spectrum GX (Perkin Elmer, USA).

### 2.4. Determination of Phenolic Acids in Propolis Extracts Containing Electrospun Nano/Microfibers

Phenolic acids (coumaric, ferulic, caffeic, and vanillic acids) and vanillin were quantified by high-performance liquid chromatography (HPLC) using Agilent 1260 Infinity capillary LC (Agilent Technologies, Inc., Santa Clara, CA, USA) with an Agilent diode array detector and performing separation on ACE C18 column (150 × 0.5 mm, 5 *μ*m particle size). The validated HPLC method was applied for quantification of the abovementioned phenolic compounds.

The mobile phase was composed of acetonitrile (solvent A) and 0.5% (v/v) acetic acid in water (solvent B). The linear elution gradient was applied from 1% to 21% of solvent A in B for 25 min. The column temperature was 25°C, the injection volume was 0.2 *μ*L, and the flow rate was 20 *μ*L/min. The absorption was determined at the wavelength of 290 nm.

All the samples were filtered through 0.20 *μ*m sterile nylon membrane filters for syringes (Sartorius Stedim Biotech GmbH, Goettingen, Germany) before HPLC analysis.

### 2.5. Study of* In Vitro* Release of Propolis Phenolic Compounds from Nano/Microfibers

The precise amounts of nano/microfibers (70–300 mg) were placed into 25 mL of aqueous acceptor medium, which was stirred periodically.* In vitro* release was determined at room temperature. The samples from the receptor solution were removed at 0.25, 0.5, 1, 2, 4, and 6 h time points and replaced with the same volume of fresh acceptor solution. Determination of phenolic compounds in the samples was performed by HPLC.

### 2.6. Assessment of Dissolution of Electrospun Nano/Microfiber Mats

The dissolution of electrospun mats produced from 8% PVP in PEE (B) and 8% PVP in PEE with 10% Ag colloidal solution (B1) was visualized by means of an inverted microscope Olympus IX71 equipped with LCAchN40xPH lens (total visual magnification, 40 × 10).

A microscopic slide with a small section of the mat was placed on a microscopic table. In order to identify changes during dissolution, mats were irrigated with distilled water drops until complete dissolution. Changes in the structure were documented after every irrigation step.

### 2.7. Release Studies of Silver Nanoparticles from Electrospun Nano/Microfibers* In Vitro* and Particle Size Determination

The precise amounts of nano/microfibers (70–300 mg) were placed into 25 mL of aqueous acceptor medium, which was stirred periodically.* In vitro* release was determined at room temperature. The samples from the acceptor solution were removed at 0.25, 0.5, 1, 2, 4, and 6 h time points and replaced with the same volume of fresh acceptor solution. All the samples were filtered through nylon membrane filters into a measuring cuvette. The mean particle size in the acceptor medium after release from nanofibers was measured by applying a dynamic light scattering technique with a Zetasizer Nano ZS particle size analyzer (Malvern, UK). Measurements were performed at the temperature of 25°C.

### 2.8. Determination of Antimicrobial Activity

The solutions and produced electrospun mats were tested for their antibacterial and antifungal activity: 8% PVP in ethanol (A), 8% PVP in PEE (B), 8% PVP in PEE containing 10% Ag colloidal solution (B1), and 8% PVP in PEE containing 20% Ag colloidal solution (B2).

The antibacterial and antifungal activity was determined* in vitro* using the agar diffusion method in Mueller-Hinton II agar medium (BBL, Cockeysville, USA). Antimicrobial activity of electrospun mats was determined on the standard bacterial (*Staphylococcus aureus* ATCC 25923,* Staphylococcus epidermidis* ATCC 12228,* Enterococcus faecalis* ATCC 29212,* Escherichia coli* ATCC 25922,* Pseudomonas aeruginosa* ATCC 27853,* Proteus vulgaris* ATCC8427,* Bacillus subtilis* ATCC 6633, and* Bacillus cereus* ATCC 11778) and fungal (*Candida albicans* ATCC 10231) strains.

### 2.9. Preparation of Standard Microorganism Cultures

Standard strains of nonsporing bacteria* Staphylococcus aureus*,* Staphylococcus epidermidis*,* Enterococcus faecalis*,* Escherichia coli*,* Pseudomonas aeruginosa*, and* Proteus vulgaris* were cultivated for 20–24 h at the temperature of 35°C in the Mueller-Hinton agar medium (Mueller-Hinton II agar, BBL, Cockeysville, USA). Bacterial suspensions were prepared from cultivated bacterial cultures in a physiological solution comparing to the 0.5 McFarland turbidity standard.

Standard cultures of spore-forming bacteria* Bacillus subtilis* and* Bacillus cereus* were cultivated for 7 days at the temperature of 35°C–37°C in the Mueller-Hinton II agar medium and washed away from the surface of broth with a sterile physiological solution. The suspensions were heated for 30 min at 70°C and diluted to obtain the concentration of spores in 1 mL ranging from 10 × 10^6^ to 100 × 10^6^ CFU. Spore suspension was kept at temperature below 4°C.

The standard fungal* Candida albicans* strain was cultivated for 20–24 h at 30°C in the Mueller-Hinton II agar medium (BBL, Cockeysville, USA). Suspension of fungal cells was prepared from strains cultivated in a physiological solution and standardized to 0.5 McFarland turbidity standard.

### 2.10. Determination of Antimicrobial Activity of Solutions Used for Electrospinning and Electrospun Mats

A, B, B1, and B2 sample solutions (1 mL) were placed into sterile Petri dishes under aseptic conditions. Liquid Mueller-Hinton agar (10 mL) preheated to 45°C was added, and both liquids were mixed. The area in the Petri dish was divided into 9 sectors, and each of them was inoculated with standardized suspensions of microbial strains and cultivated for 20–24 h at 35°C. Growth of tested strains indicated no antimicrobial activity.

Positive control was performed by addition of 7% ethanol aqueous solution (1 mL) to standard microorganism cultures in the Mueller-Hinton II agar medium. Negative control was performed by addition of 15% ethanol aqueous solution (1 mL) to standard microorganism cultures in the Mueller-Hinton II agar medium.

Samples of electrospun mats were prepared by cutting the electrospun mats to 30 × 30 mm sheets. All the samples were sterilized with ultraviolet radiation. Microbial cell suspensions at the density of 1 × 10^8^ CFU/mL were used for inoculation of Mueller-Hinton agar media. The samples of sterilized electrospun mats were placed on the surface of Mueller-Hinton agar inoculated with microbial cultures under aseptic conditions. The samples were incubated at 36°C for 24 h for bacteria and at 30°C for 24 h for fungi. The antimicrobial activity of samples was determined by formation the sterile zone around the inserted sample.

## 3. Results and Discussion

### 3.1. The Structure of Electrospun Mats

The structure of electrospun mats is affected mostly by technological parameters such as applied voltage and distance between electrodes, and especially by properties of the polymer solution, including concentration, surface tension, and nature of solvent [29]. In this study, the applied voltage was 30 kV, and the distance between electrodes was 130 mm during all electrospinning procedures. The concentration of electrospun solution, nature of solvent (ethanol or PEE), and composition of electrospun solution (without or with Ag NPs) were parameters that varied.

**Figure 2:**
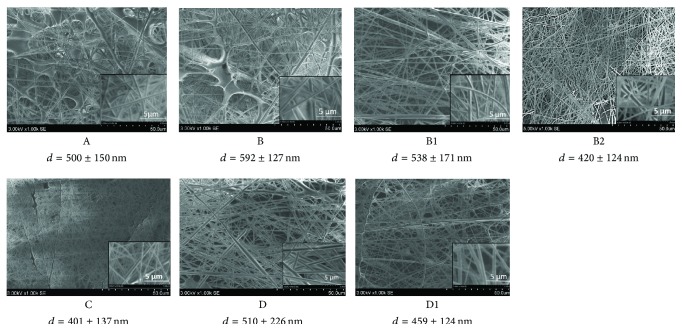
SEM images of electrospun nano/microfibers. Magnification ×1000, scale 50 *μ*m, and magnification ×10 000, scale 5 *μ*m. The values are the mean diameter *d* of nano/microfibers with standard deviation.

**Figure 3:**
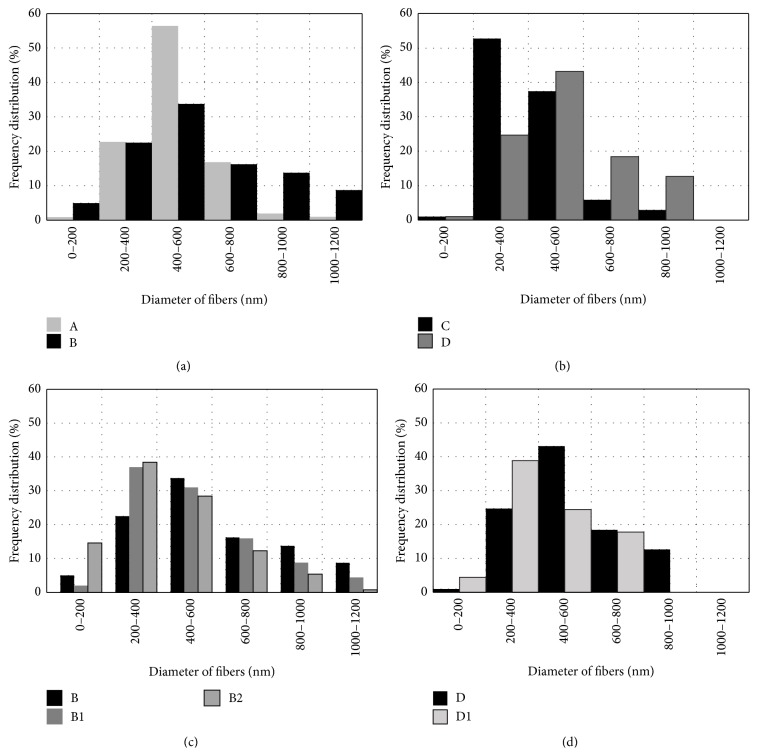
Distribution of electrospun PVP nano/microfibers, produced from 8% PVP solution (a), 6% PVP solution (b), and 8% PVP solution with Ag NPs (c), and 6% PVP solution with Ag NPs (d) by diameter.

Figure 2 shows SEM images of electrospun nano/microfiber mats. Analysis of the structure of electrospun nano/microfibers demonstrated the formation of thicker fibers and spots of polymer solution when concentration of PVP in solution was 8% (samples A and B). These defects were documented less frequently when Ag NPs were included into the electrospun solution (samples B1 and B2) and when the concentration of PVP was 6% (samples C and D). The distribution of electrospun PVP nano/microfibers by the diameter is depicted in Figure 3. Thicker fibers were formed from PVP in the PEE solution, though the viscosity of PVP solutions in ethanol and PEE was similar. The viscosity of 8% and 6% PVP solutions was 150–160 and 94–110 mPa·s, respectively. The diameter of the produced fibers was up to 600 nm in 79% of the cases when 8% PVP in ethanol solution (A) was used and 61% of the cases when 8% PVP in PEE (B) was used (Figure 3(a)). The similar tendency was observed when the concentration of PVP in solutions was 6% (Figure 3(b)): 91% and 69% of the fibers, produced from 6% PVP solution in ethanol (C) and PEE (D), respectively, had a diameter up to 600 nm.

Inclusion of Ag NPs into the electrospun PVP solution was associated with the formation of thinner fibers (Figures 3(c) and 3(d)). It was determined that 53%, 39%, and 28% of the fibers had a diameter up to 400 nm when electrospun PVP solution contained 20% Ag colloidal (B2), 10% Ag colloidal (B1), or no Ag colloidal (B) solutions, respectively. Addition of 10% Ag colloidal solution to 6% PVP solution (D1) resulted in an increased percentage of fibers with a diameter up to 400 nm from 26% (D) to 43% (D1). The presence of Ag NPs in the polymer solution increased its conductivity and charge density thus leading to increased columbic forces [21]. It resulted in polymer jet splitting and formation of thinner fibers with fewer defects. Our findings are in agreement with the published data of similar studies [20, 21].

Thinner fibers were electrospun using 6% PVP solution, and this could be explained by lower viscosity of solution. When the concentration of PVP in ethanol solution was 6% (C), 53% of the fibers had a diameter ranging from 200 to 400 nm. When the concentration of PVP in the solution was 8% (A), 57% of the produced fibers had a diameter of 400–600 nm.

Inclusion of colloidal Ag NPs into the electrospun solution of PVP in PEE resulted in the formation of thinner and more uniform nano/microfibers. The uniformity of nano/microfiber mats increased when the concentration of PVP in the solution was 6%.

**Figure 4:**
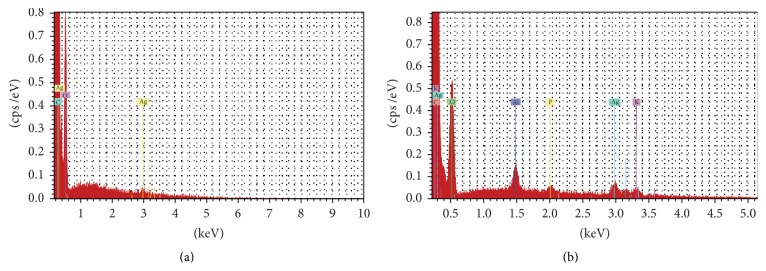
EDS spectra of electrospun PVP mats containing PEE and 10% (a) or 20% (b) colloidal Ag.

Elemental analysis of PVP mats containing PEE and Ag NPs was performed by SEM-EDS. Spectra of 8% PVP in PEE containing colloidal Ag solution are displayed in Figure 4. EDS analysis confirmed the presence of Ag in electrospun mats produced from PVP/propolis/Ag NP solution.

### 3.2. FT-IR Analysis

**Figure 5:**
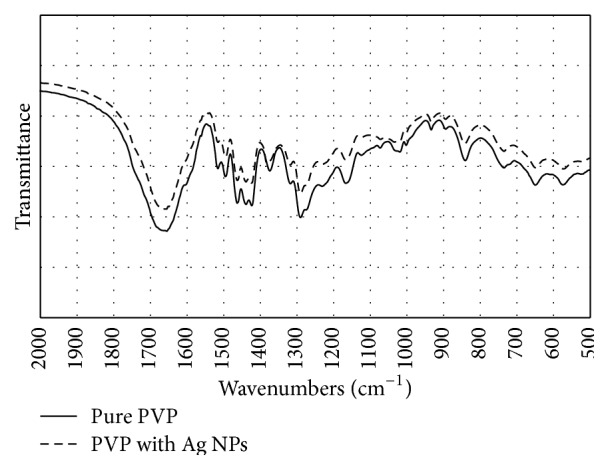
FT-IR spectra of PVP and PVP with Ag NPs.

FT-IR spectroscopy is considered as an efficient tool to analyze the solid phase structure as FT-IR spectra can provide additional information about possible interactions between PVP, PEE, and Ag NPs in the produced electrospun mats (Figure 5).

The FT-IR characteristic absorption peaks of PVP nano/microfibers were observed at 1665, 1440, 1370, and 1290 cm^−1^, which are attributed to the vibration of the carbonyl group (C-O), O-H bending, lactone structure, and -C-N- stretching, respectively [30]. Analysis of Ag NPs in PVP demonstrated the presence of molecular interactions between PVP and Ag NPs [30]. The shift of the bands was not identified when a possible interaction of Ag NPs and PVP was analyzed; therefore, no strong molecular interaction between PVP and Ag NPs was confirmed. The comparison of FT-IR spectra of PVP containing Ag NPs with single PVP spectra revealed no significant differences that could indicate the presence of interactions between PVP and Ag NPs in produced nano/microfibers.

### 3.3. *In Vitro* Release Study of Propolis Phenolic Acids from Nano/Microfibers

The quality of produced nano/microfibers was defined by release profile of propolis phenolic compounds and Ag NPs. Biopharmaceutical characterization of nano/microfiber mats was performed by determining the dissolved amounts of vanillic, caffeic, p-coumaric, and ferulic acids and vanillin thus evaluating possible complex interactions of phenolic compounds with a fiber-forming polymer and incorporated Ag NPs. The released fraction of phenolic acids (vanillic, caffeic, p-coumaric, and ferulic) and vanillin differed depending both on the concentration of PVP and presence of Ag NPs in the fibers (Table 2). Efficient release was demonstrated only in case of vanillic acid from PVP nano/microfibers produced using 8% PVP solution in PEE and for vanillic, caffeic, and ferulic acids when fibers were produced from 6% PVP solution in PPE containing 10% Ag colloidal solution. In all the remaining cases, the released fractions of phenolic acids and vanillin ranged from 32.2% to 82.9% thus indicating slower release of phenolic compounds from nano/microfibers during dissolution testing.

Unexpectedly high release of propolis phenolic compounds was identified for nano/microfibers produced by electrospinning from 6% PVP solution containing 10% colloidal Ag. All amount of vanillic, caffeic, and ferulic acids and around 80% of p-coumaric acid and vanillin present in nano/microfibers were released in 6 h. The release of the same phenolic compounds from nano/microfibers without colloidal Ag was significantly lower during the same time interval of dissolution testing.

### 3.4. Dissolution of Electrospun Nano/Microfiber Mats

The mean size of Ag NPs in the initial solution was 97.47 ± 0.512 nm. The dilution had a negligible effect on the mean particle size as it decreased to 88.76 nm after 100-fold dilution of the tested sample and to 81.91 nm after 5000-fold dilution. These results imply the presence of Ag NP agglomerates, and the mean size of Ag NP agglomerates could be affected by the concentration of Ag in the liquid phase.

**Figure 6:**
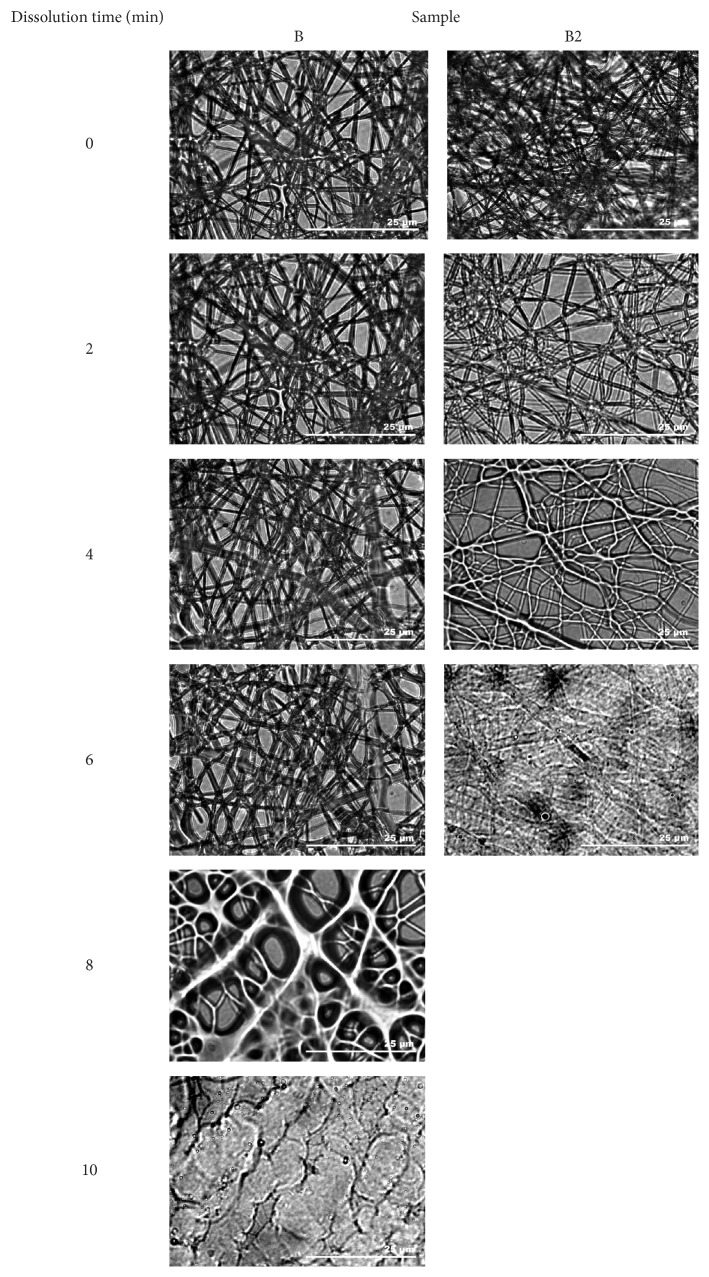
Disintegration and dissolution of PVP-based nano/microfiber mats containing PEE and Ag NPs.

The disintegration and dissolution process of electrospun mats was analyzed in order to obtain general information on the behavior of PEE and colloidal Ag containing nano/microfibers in the presence of aqueous fluids. This should contribute to the understanding of mechanisms involved in the release of propolis phenolic acids and Ag NPs from electrospun PVP nano/microfibers. The changes in the mat structure during the dissolution process are depicted in Figure 6. The visual disintegration and dissolution processes of nano/microfibers mats lasted up to 10 min. Water disrupted the fragments of nano/microfibers mats formulated using 8% PVP in PEE with 20% colloidal Ag solution (B2) and containing propolis extract components and Ag NPs immediately after contact with water. After coming into contact with water, nano/microfiber strands started dissolving and dissolved completely in up to 5 min as only fragments of primary nano/microfibers could be identified. The dissolution of nano/microfibers mats formulated using 8% PVP solution in PEE (B) and containing only PEE occurred less intensely. In the latter case, the dissolution of nano/microfibers required more water, it was slower, and more different stages of dissolution were observed. These results could be related to the different structure of electrospun mats. Mats containing Ag NPs (B2) were composed of more uniform and thinner nano/microfibers.

The release and presence of Ag NPs in the acceptor liquid during mat dissolution testing were confirmed by determining the size of dominating Ag NPs.  Table 3 depicts the mean nanoparticle size measured at different time points in different samples. The results showed that the mean size of Ag NPs present in the acceptor phase increased with time for the samples produced from PVP (8%) in PEE with 10% (B1) and 20% (B2) Ag colloidal solution and from PVP (6%) in PEE with 10% Ag colloidal solution (D1).

The application of the light scattering technique for characterization of Ag NPs provided the data of fractional distribution of NPs by the mean particle size in the aqueous medium following PVP nano/microfiber dissolution. The total number of Ag NPs in the aqueous medium increased when dissolution and release characteristics of nano/microfibers containing 10% Ag colloidal solution were evaluated. For these nano/microfibers, it was determined that the fraction of particles with a smaller diameter increased during 6 h period of observation. Dissolution testing of PVP nano/microfibers containing 20% colloidal Ag showed a decreased number of particles and an increased mean particle size in the aqueous medium. The mean particle size varied in the individual fractions during nano/microfiber dissolution testing, but generally it resulted in an increase in the mean particle size from 44% to 147%.

### 3.5. Antibacterial Activity of Electrospun Solutions

The antibacterial and antifungal activity of electrospun solutions was tested on standard microbial strains* in vitro*, and the results are presented in Table 4. There was no antimicrobial activity of PVP in the aqueous ethanol solution (A). PVP in the PEE solution (B) demonstrated antibacterial properties against* Enterococcus faecalis*,* Bacillus subtilis*,* Bacillus cereus*, and* Candida albicans*. Antibacterial and antifungal activity of solutions was determined against all tested microorganism strains when PVP/PEE solutions containing Ag NPs (B1 and B2) were applied. Similar antibacterial activity against all tested microorganism strains was determined after application of electrospun mats containing colloidal Ag NPs. These results confirm that the electrospinning technique had no effect on either biologically active components from PEE or antimicrobial activity of Ag NPs included in nano/microfibers.

Antimicrobial activity testing also confirmed the efficient release of propolis phenolic compounds and Ag NPs from formulated nano/microfibers, as demonstrated similar pattern of inactivation of microorganism strains to that of PVP solutions in PEE and containing Ag NPs.

## 4. Conclusions

The results of present study confirmed the possibility to incorporate biological active compounds of PEE into PVP mats by the electrospinning technique. Produced nano/microfiber mats exhibited fast release of propolis phenolic compounds and it may be used in the further development of products stimulating wound healing. Inclusion of PEE into the electrospun PVP solution demonstrated no significant influence on the structure of electrospun mats. Additional inclusion of Ag NP colloidal solution into a mixture of PVP and PEE led to the formation of homogeneous and relatively thin nano/microfibers. Solutions of PVP with PEE and electrospun mats demonstrated low antimicrobial activity and affected the growth of* Bacillus subtilis* ATCC 6633 and* Bacillus cereus* ATCC 11778. Inclusion of Ag NPs into electrospun solutions of PVP with PEE and into produced mats increased their antimicrobial activity, and this was demonstrated by the inhibition of growth of* Staphylococcus aureus* ATCC 25923,* Staphylococcus epidermidis* ATCC 12228,* Enterococcus faecalis* ATCC 29212,* Escherichia coli* ATCC 25922,* Pseudomonas aeruginosa* ATCC 27853,* Proteus vulgaris* ATCC 8427,* Bacillus subtilis* ATCC 6633,* Bacillus cereus* ATCC 11778, and* Candida albicans* ATCC 10231 strains. The results demonstrated suitability of the electrospinning technique for the production of fast dissolving antimicrobial nano/microfiber mats with propolis phenolic compounds and Ag NPs.

## Figures and Tables

**Table 1:** Composition of electrospun PVP solutions.

Code of sample	Composition of electrospun solutions
A	PVP (8%) in ethanol
B	PVP (8%) in PEE
C	PVP (6%) in ethanol
D	PVP (6%) in PEE
B1	PVP (8%) in PEE with 10% Ag colloidal solution
B2	PVP (8%) in PEE with 20% Ag colloidal solution
D1	PVP (6%) in PEE with 10% Ag colloidal solution

**Table 2:** Maximum quantities of phenolic acids released from nano/microfibers in 6 h.

Sample code	Composition of electrospun solutions	Released quantity (%) of phenolic compounds				
		Vanillic acid	Caffeic acid	p-Coumaric acid	Ferulic acid	Vanillin
B	PVP in PEE (*C* = 8%)	99.9	52.1	42.1	58.6	74.3
B1	PVP in PEE (*C* = 8%) with 10% Ag colloidal solution	71.1	48.6	38.0	49.3	53.6
B2	PVP in PEE (*C* = 8%) with 20% Ag colloidal solution	66.9	32.2	37.9	43.0	45.4
D	PVP in PEE (*C* = 6%)	65.0	50.7	35.6	47.1	48.9
D1	PVP in PEE (*C* = 6%) with 10% Ag colloidal solution	100.0	100.0	82.9	100.0	79.1

**Table 3:** Size of Ag NPs released from nano/microfibers.

Sample	Particle size, mean ± SD, nm					
	0.25 h	0.5 h	1 h	2 h	4 h	6 h
B1	37.53 ± 0.75	42.08 ± 0.50	41.34 ± 0.07	61.87 ± 1.02	NA	65.15 ± 0.87
B2	38.62 ± 2.56	38.71 ± 0.28	41.37 ± 1.08	52.64 ± 1.09	50.32 ± 0.19	55.62 ± 0.67
D1	30.85 ± 0.58	33.77 ± 0.15	37.45 ± 0.35	49.98 ± 0.68	71.95 ± 1.06	76.34 ± 1.09

NA, not available.

**Table 4:** Antimicrobial activity of electrospun solutions and nano/microfiber mats.

Number	Standard strains of microorganisms	Electrospun solutions				Produced mats			
		A	B	B1	B2	A	B	B1	B2
1	*Staphylococcus aureus* ATCC 25923	−	*−*	*+*	*+*	*−*	*−*	*+*	*+*
2	*Staphylococcus epidermidis* ATCC 12228	*−*	*−*	*+*	*+*	*−*	*−*	*+*	*+*
3	*Enterococcus faecalis* ATCC 29212	*−*	*+*	*+*	*+*	*−*	*−*	*+*	*+*
4	*Escherichia coli* ATCC 25922	−	−	+	+	−	−	+	+
5	*Pseudomonas aeruginosa* ATCC 27853	*−*	*−*	*+*	*+*	*−*	*−*	*+*	*+*
6	*Proteus vulgaris* ATCC 8427	*−*	*−*	*+*	*+*	*−*	*−*	*+*	*+*
7	*Bacillus subtilis* ATCC 6633	*−*	*+*	*+*	*+*	*−*	*+*	*+*	*+*
8	*Bacillus cereus* ATCC 11778	−	+	+	+	−	+	+	+
9	*Candida albicans* ATCC 10231	−	+	+	+	−	+	+	+

“−” no antibacterial activity determined, “+” antibacterial activity determined.
